# The burdens attributable to primary headache disorders in children and adolescents in Iran: estimates from a schools-based study

**DOI:** 10.1186/s10194-024-01789-0

**Published:** 2024-05-27

**Authors:** Mansoureh Togha, Pegah Rafiee, Faraidoon Haghdoost, Shahram Rafie, Seyed Mohammad Hasan Paknejad, Sepideh Amouian, Tayyar Şaşmaz, Derya Kale, Derya Uluduz, Timothy J. Steiner

**Affiliations:** 1 Headache Department, Iranian Center of Neurological Researches, Institute of Neuroscience, Tehran University of Medical Sciences, Tehran, Iran; 2 Neurology Ward, School of Medicine, Sina Hospital, Tehran University of Medical Sciences, Tehran, Iran; 3 Department of Clinical Nutrition and Dietetics, Faculty of Nutrition Sciences and Food Technology, National Nutrition and Food Technology Research Institute, Shahid Beheshti University of Medical Sciences, Tehran, Iran; 4 The George Institute for Global Health, University of New South Wales (UNSW), Sydney, Australia; 5 Department of Neurology, School of Medicine, Ahvaz Jundishappur University of Medical Sciences, Ahvaz, Iran; 6 Neonatal and Children’s Health Research Center, Golestan University of Medical Sciences, Gorgan, Iran; 7 Pediatric Neurology Department, Faculty of Medicine, Golestan University of Medical Sciences, Gorgan, Iran; 8 Department of Public Health, Mersin University School of Medicine, Mersin, Turkey; 9 Neurology Department, Cerrahpaşa School of Medicine, Istanbul University, Istanbul, Turkey; 10 Department of Neuromedicine and Movement Science, Norwegian University of Science and Technology, Edvard Griegs gate, Trondheim, Norway; 11 Department of Neurology, University of Copenhagen, Copenhagen, Denmark; 12 Division of Brain Sciences, Imperial College London, London, UK

**Keywords:** Child and adolescent headache, Migraine, Tension-type headache, Undifferentiated headache, Medication-overuse headache, Epidemiology, Burden of headache, Schools-based study, Educational policy, Health policy, Iran, Eastern Mediterranean Region, Global Campaign against Headache

## Abstract

**Background:**

We recently found headache disorders to be highly prevalent among children (aged 6–11 years) and adolescents (aged 12–17) in Iran (gender- and age-adjusted 1-year prevalences: migraine 25.2%, tension-type headache 12.7%, undifferentiated headache [UdH] 22.1%, probable medication-overuse headache [pMOH] 1.1%, other headache on ≥ 15 days/month [H15+] 3.0%). Here we report on the headache-attributed burden, taking evidence from the same study.

**Methods:**

In a cross-sectional survey, following the generic protocol for the global schools-based study led by the Global Campaign against Headache, we administered the child and adolescent versions of the Headache-Attributed Restriction, Disability, Social Handicap and Impaired Participation (HARDSHIP) structured questionnaire in 121 schools, purposively selected to reflect the country’s diversities. Pupils self-completed these in class, under supervision. Headache diagnostic questions were based on ICHD-3 criteria but for the inclusion of UdH (defined as mild headache with usual duration < 1 h). Burden enquiry was across multiple domains.

**Results:**

The analysed sample (*N* = 3,244) included 1,308 (40.3%) children and 1,936 (59.7%) adolescents (1,531 [47.2%] male, 1,713 [52.8%] female). The non-participating proportion was 3.4%. Mean headache frequency was 3.9 days/4 weeks, and mean duration 1.8 h. Estimated mean proportion of time in ictal state was 1.1% (1.4% for migraine, 16.5% for pMOH). Symptomatic medication was consumed on a mean of 1.6 days/4 weeks. Lost school time averaged 0.4 days/4 weeks overall (2%, assuming a 5-day week), but was eleven-fold higher (4.3 days; 22%) for pMOH. For most headache types, days of reported limited activity were several-fold more than days lost from school (45% for pMOH, 25% for other H15+). Almost one in 12 parents (7.9%) missed work at least once in 4 weeks because of their son’s or daughter’s headache. Emotional impact and quality-of-life scores reflected these measures of burden.

**Conclusions:**

Headache, common in children and adolescents in Iran, is associated with symptom burdens that may be onerous for some but not for most. However, there are substantial consequential burdens, particularly for the 1.1% with pMOH and the 3.0% with other H15+, who suffer educational disturbances and potentially major life impairments. These findings are of importance to educational and health policies in Iran.

## Introduction

Headache disorders in adulthood, the third-highest cause of years lived with disability (YLDs) worldwide [1, 2], commonly have their onset in pre-adult years [3]. Multiple studies have found that migraine and tension-type headache (TTH) are already prevalent among children (aged 6–11 years) and adolescents (12–17 years) [4–10]. All of these studies also recognized “undifferentiated headache” (UdH) – mild headache of short duration (< 1 h) – as, probably, an immature form of migraine or TTH while not meeting diagnostic criteria for either [4]. In several of these studies, UdH was more prevalent than migraine [4–6], raising an intriguing and potentially important question: can early (pre-adult) intervention attenuate the later (adult) expression of migraine? The answer to this remains unknown, but these studies also noted that medication-overuse headache (MOH) was far from rare in these age groups, particularly among adolescents, although less prevalent than among adults in the same countries, where this had been assessed [11–14]. MOH, at least, is modifiable if recognized [15].

Whatever opportunity there might be to mitigate it in later life, headache-attributed burden among children and adolescents represents a loss of health among these young people. It impairs their quality of life, disturbs their participation in daily life and – most importantly – has the potential to undermine their schooling [16]. The consequences are not only serious but also enduring. For these reasons, studies of headache-attributed burden are as necessary in children and adolescents, informing both health and educational policies, as they are in adults.

In Iran, we have already reported the prevalence of headache disorders in these age groups, gathering data in a nationwide schools-based enquiry [7]. This survey, conducted as part of a global study [17] under the auspices of the Global Campaign against Headache [18, 19], was the first such study from Eastern Mediterranean Region. It found, as had the studies elsewhere [4–6, 8–10], high 1-year prevalences of episodic headache disorders: migraine (25.2%), TTH (12.7%) and UdH (22.1%). Headache on ≥ 15 days/month (H15+) was reported by 4.1%, and identified as probable MOH (pMOH) in 1.1% and as “other H15+” in 3.0% [7]. All of these were strongly age-related: higher among adolescents than among children. Here we present data on multiple components of the burdens attributed to these disorders, which were collected in the same study. They add to knowledge of the global burden of headache [2] and to our understanding of headache disorders as they develop in young people, and they inform health and educational policies in Iran.

## Methods

The study was a cross-sectional survey conducted in schools in accordance with the generic protocol [17]. The methodology, fully described elsewhere [7], is summarized here.

### Ethics and approvals

The study was approved by the Ethics Committee of Tehran University of Medical Sciences, Tehran. Managers and teachers at the selected schools agreed to their participation. Informed consents were obtained from participants and their parents.

Data were gathered anonymously.

### Sampling and enquiry

We sampled from 121 schools, purposively selected to reflect the country’s geographical, genetic, cultural and socioeconomic diversities [7]. In these schools, all pupils aged 6–17 years, from all classes, were invited to participate. We aimed for *N* > 3,000 in accordance with published guidelines [20].

Enquiry used the child and adolescent versions of the Headache-Attributed Restriction, Disability, Social Handicap and Impaired Participation (HARDSHIP) structured questionnaire [17], translated into the Persian language [21]. Pupils aged 6–17 years completed these (45 questions), in class and within a single class period of 30–40 min, under supervision of the teacher or an investigator. Younger children, and those unable to read well, were given necessary assistance in understanding and appropriately answering the questions.

Diagnostic questions were based on ICHD-3 criteria [22], except for UdH. H15 + was diagnosed as pMOH when associated with reported overuse of symptomatic medication (on ≥ 14 days in the preceding 4 weeks) and otherwise as “other H15+”. Among pupils with headache on < 15 days/month, UdH was identified by the two characteristics of mild intensity and short duration (< 1 h) [17]. Among those remaining, diagnoses of definite migraine, definite TTH, probable migraine and probable TTH, in this strict order, were made algorithmically [7, 17]. For analysis of attributed burden, definite migraine and probable migraine were combined, as were definite and probable TTH.

### Headache-attributed burden

Enquiry included symptom burden (frequency, and usual duration and intensity of headache episodes), acute medication use, time lost from school and from other activities, and time lost by parents from their own work while tending to their son’s or daughter’s headache [17]. A validated subset of questions in HARDSHIP included selected (headache-relevant) questions from KINDL® [23], addressing concentration, emotional impact and quality of life (QoL) [17].

### Data management and analyses

All data were entered into SPSS, with errors corrected by comparing the SPSS dataset with the original questionnaires.

Headache frequency (F) and medication intake were counted as days in the preceding 4 weeks. Headache intensity was expressed on a scale of 1–3, equating to “mild”, “moderate” and “severe”. Duration of episodes (D) was reported categorically, in hours as < 1, 1–2, 2–4 or > 4. We analysed these according to the mid-points of each (0.5, 1.5, 3 and 8 h respectively, assuming a range of 4–12 h for the last). We calculated the proportion of time in ictal state (pTIS) as (F/28*D/24). We calculated headache-attributed lost health at individual level as pTIS*DW, where DW was the disability weight (DW) attributed to the ictal state of each headache type in the Global Burden of Disease (GBD) study 2013 [24, 25] (it should be noted that DWs reflect lost health in a broad sense rather than disability [26–28]). We estimated population-level lost health [24] for each headache type as the product of mean individual lost health and prevalence. Lost school time because of headache was counted in days of absence in the preceding 4 weeks, with partial absences (leaving school early) as half-days. Separately, we counted reported days of limited activity (“I could not do things I wanted to because of my headaches”). We counted pupils reporting a lost school day because of headache yesterday (HY), and estimated predicted values of lost school yesterday as the product of the number affected by headache and mean reported schooldays (divided by 20) lost per pupil with headache over the preceding 4 weeks. Parents’ lost time from their own work during the same period was reported by pupils as “yes” or “no”, regardless of the quantity of time. Responses to questions addressing concentration, emotional impact and QoL were all on a 4-point Likert scale (“never”, “sometimes”, “often”, “always”), scored as 0–3. We summed the scores to generate an emotional impact score (including concentration; potential range 0–18, high being adverse) and a QoL score (0–36, low being adverse) [17].

We expressed proportions as %, using chi-squared for comparisons. We treated all other variables as continuous, using descriptive statistics (means and standard deviations [SDs]), and compared these data using t-test and ANOVA when they were distributed normally, and otherwise Mann-Whitney U test and Kruskal-Wallis test with *post hoc* Dunn test.

We considered *p* < 0.05 to be significant.

## Results

Of 3,357 potential participants, 14 (8 males, 6 females) did not take part (five absent on the day and nine declining to participate). Questionnaires of a further 99 were insufficiently completed for analysis (> 50% of responses missing). Therefore, the analysed sample were *N* = 3,244 (participating proportion 96.6%). Of these, 1,308 (40.3%) were children, 1,936 (59.7%) were adolescents (overall mean age 12.3 [± 3.2] years, median 12.0 years), 1,531 (47.2%) were male and 1,713 (52.8%) were female.

### Symptom burden

Table 1 shows headache frequency, duration and intensity by headache type, along with estimated pTIS. For later analyses, frequency, duration and pTIS are shown for each age group for migraine, TTH and pMOH.

Overall mean headache frequency was 3.9 days/4 weeks. Among episodic headaches, migraine had the highest frequency (3.7 days/4 weeks). H15 + was, of course, more frequent, with 20.9 days/4 weeks reported for pMOH (Table 1). Duration of headache was not normally distributed but, judged nonetheless by their means, episodes of migraine (2.6 h) and TTH (1.8 h) were generally short-lasting, with, as expected, UdH (0.5 h) of shorter duration than either (*p* < 0.001) (Table 1). pMOH (5.3 h) was notably longer-lasting (*p* < 0.01). Estimated mean pTIS was correspondingly low overall mean (1.1%) but, for pMOH, reached 16.5% (Table 1).

Mean headache intensity (1.6 overall) was greater for migraine (2.1: moderate) than for TTH (1.4: mild-to-moderate; *p* < 0.001). UdH, always mild by definition, was rated less intense than all other headache types (all *p* < 0.001) (Table 1).

**Table 1:** Symptom burden by headache type and overall (*N* = 3,244)

Headache type	Pupils affected *n*	1-year prevalence^1^ %	Mean frequency days/4 weeks	Mean duration hours	Estimated proportion of time in ictal state %	Mean intensity (scale 1–3)^2^
**Episodic headaches**						
Migraine	910		3.7 ± 3.1	2.6 ± 2.4	1.4	
children adolescents	243 667	25.2%	2.9 ± 2.9 4.0 ± 3.1	2.4 ± 2.3 2.7 ± 2.4	1.0 1.6	2.1 ± 0.6
Tension-type headache	435		2.7 ± 2.7	1.8 ± 1.5	0.7	
children adolescents	130 305	12.7%	1.8 ± 2.1 3.0 ± 2.9	1.7 ± 1.5 1.8 ± 1.5	0.4 0.8	1.4 ± 0.6
Undifferentiated headache	750	22.1%	1.8 ± 2.3	0.5 ± < 0.1	0.1	1.0 ± < 0.1
**Headache on ≥ 15 days/month**						
Probable medication-overuse headache	41		20.9 ± 4.3	5.3 ± 3.2	16.5	
children adolescents	5 36	1.1%	23.2 ± 4.4 20.5 ± 4.2	5.4 ± 3.1 5.3 ± 3.2	18.6 16.1	2.5 ± 0.6
Other headache on ≥ 15 days/month	121	3.0%.	18.5 ± 4.3	2.9 ± 2.6	8.0	2.2 ± 0.6
**All headache** ^**3**^	2,301	65.4%	3.9 ± 5.2	1.8 ± 2.1	1.1	1.6 ± 0.7

Means are presented ± SDs; ^1^gender- and age-adjusted (data from [7]); ^2^equating 1–3 to the reported categories “mild”, “moderate” and “severe”, and treating as continuous data; ^3^includes unclassified headache

**Table 2:** Symptomatic medication use by headache type and overall

Headache type	Preceding week		Preceding 4 weeks	
	*n* ^1^	days (mean ± SD)	*n* ^1^	days (mean ± SD)
**Episodic headaches**				
Migraine	884	0.7 ± 1.1	910	1.7 ± 2.3
Tension-type headache	426	0.4 ± 0.8	435	1.1 ± 1.7
Undifferentiated headache	732	0.2 ± 0.6	750	0.5 ± 1.3
**Headache on ≥ 15 days/month**				
Probable medication-overuse headache	36	4.1 ± 1.9	41	18.0 ± 3.3
Other headache on ≥ 15 days/month	118	1.4 ± 1.6	121	4.3 ± 4.3
**All headache** ^**2**^	2,239	0.6 ± 1.1	2,301	1.6 ± 3.2

^1^Values of n vary because not all pupils responded to both questions; ^2^includes unclassified headache

**Table 3:** Headache-attributed lost health by headache type (*N* = 3,244)

Headache type	Disability weight (from [25])	Prevalence (%) (from [7])	pTIS (%) (from Table 1)	Lost health (%)	
				Individual^1^ mean	Population^2^
Migraine		25.2	1.4	0.6	0.16
children adolescents	0.441	18.6 34.5	1.0 1.6	0.4 0.7	0.08 0.24
Tension-type headache		12.7	0.7	0.03	0.004
children adolescents	0.037	9.9 15.8	0.4 0.8	0.02 0.04	0.002 0.005
Probable medication-overuse headache		1.1	16.5	3.7	0.04
children adolescents	0.223	0.4 1.9	18.6 16.1	4.2 3.6	0.02 0.07

pTIS: proportion of time in ictal state; ^1^calculated as the product of pTIS and disability weight; ^2^calculated as the product of mean individual lost health and prevalence

### Medication use

Reported symptomatic medication intake for the preceding week and 4 weeks is shown in Table 2, with consistent responses to the two enquiries. Comparison with Table 1 reveals that, for the episodic headaches, medication days were fewer than headache days, the former averaging < 2 per month for both migraine and TTH and significantly fewer for UdH (*p* < 0.001). Medication was, of course, taken much more frequently for pMOH than for all other headache types, including other H15+ (all *p* < 0.001) (Table 2).

**Table 4:** Pupils’ lost time from school and other activity because of headache in the preceding 4 weeks, by headache type and overall, and by age group

Headache type	Headache frequency (from Table 1) days/4 weeks	Days of lost school^1^	Days of limited activity^1^
		days/4 weeks/affected pupil (mean ± SD)	
**Episodic headaches**			
Migraine	3.7	0.4 ± 1.0	1.9 ± 2.4
children	2.9	0.4 ± 0.9	1.3 ± 1.9
adolescents	4.0	0.3 ± 1.0	2.1 ± 2.6
Tension-type headache	2.7	0.2 ± 0.8	1.2 ± 1.8
children	1.8	0.2 ± 0.7	0.5 ± 0.9
adolescents	3.0	0.2 ± 0.8	1.4 ± 2.0
Undifferentiated headache		0.2 ± 0.8	0.6 ± 1.3
children	1.8	0.2 ± 0.6	0.4 ± 1.0
adolescents		0.5 ± 1.2	0.8 ± 1.5
**Headache on ≥ 15 days/month**			
Probable medication-overuse headache	20.9	4.3 ± 8.1	12.6 ± 8.6
children	23.2	11.7 ± 14.5	18.6 ± 7.9
adolescents	20.5	3.2 ± 6.4	11.7 ± 8.4
Other headache on ≥ 15 days/month	18.5	0.8 ± 1.8	7.0 ± 6.8
children		0.7 ± 1.6	4.4 ± 5.9
adolescents		0.8 ± 1.9	7.3 ± 6.8
**All headache**	3.9	0.4 ± 1.5	1.8 ± 3.3
children		0.4 ± 1.7	0.9 ± 2.4
adolescents		0.4 ± 1.5	2.2 ± 3.6

^1^Lost school time has a denominator of 20, limited activity 28 (see text for explanation)

### Headache-attributed lost health

Table 3 shows lost-health estimates for migraine, TTH and pMOH, calculated at individual and population levels (GBD 2013 allocated DWs to the ictal states of these three headache disorders only [24]). In these estimates, 0% indicates no detriment to health and 100% indicates total loss (a health state valued as no better than being dead [24, 25]).

At individual level, pMOH was associated with by far the most lost health (6-fold among all ages, compared with migraine). However, at population level, with prevalence factored in, migraine overtook pMOH by a distance (four-fold among all ages) (Table 3). At this level, the degrees of lost health from all three headache types (migraine, TTH and pMOH) were about three-fold greater among adolescents than children, a consequence of higher prevalence and, for all but pMOH, greater mean pTIS among adolescents (Table 3). However, only migraine was responsible for > 0.1% of lost health distributed across the entire population of children and adolescents.

### Headache-attributed lost time

Table 4 shows pupils’ headache-attributed lost time from school and from other activities, with headache frequencies from Table 1 included for comparison.

Lost school time per pupil with headache averaged 0.4 days/4 weeks overall (i.e., 0.1 day/week, or 2% assuming a 5-day week). Standing out among headache types was pMOH, with lost school time of 4.3 days/4 weeks (i.e., > 1 day/week, or 22%). Age-related differences were minor except for pMOH, to which children (*n* = 5) attributed greater losses than adolescents (11.7 vs. 3.2 days/4 weeks; *p* = 0.051; Table 4).

Days of reported limited activity were several-fold more (overall, 1.8 days/4 weeks) than days lost from school, even allowing for the difference in denominator (28 for the former, 20 for the latter). Generally, about half of days with headache (one third for UdH) were reportedly associated with limited activity, although variance was high for all headache types (Table 4). Again, pMOH stood out, along with other H15+, affecting 45% (12.6/28) and 25% (7.0/28) of days respectively. Losses attributed to pMOH were numerically greater among children than adolescents, but the reverse was the case for all other headache types (Table 4).

Lost school days attributed to HY are shown in Table 5, along with the previously published proportions reporting HY [7]. Column one identifies pupils by their diagnosed headache type, with the assumption that HY was of that type, although this might not always have been the case. The probability of missing a school day because of HY was 6.3% overall, varying from < 1% for UdH to 26.5% for pMOH. Table 5 compares actual lost school yesterday with predictions based on the 4-week (recalled) data of Table 4 (column 3). Thus, for example, the prediction for all headache was (0.4/20)*2,279 = 46. Actual values closely matched predicted values except for UdH (lower) and other H15+ (higher), both with small absolute numbers.

**Table 5:** Pupils’ lost school time yesterday because of headache yesterday (HY), by headache type and overall

Diagnosed headache type	Pupils affected *n* ^1^	Proportion reporting HY (from [7]) *n* (% of those with headache type)	Missed school day yesterday *n* (% of those reporting HY)	
			Actual	Predicted
**Episodic headaches**				
Migraine	901	315 (35.0)	16 (5.0)	18 (5.7)
Tension-type headache	435	92 (21.1)	3 (3.3)	4 (4.7)
Undifferentiated headache	737	118 (16.0)	1 (0.8)	7 (6.2)
**Headache on ≥ 15 days/month**				
Probable medication-overuse headache	41	34 (82.9)	9 (26.5)	9 (25.9)
Other headache on ≥ 15 days/month	121	101 (83.5)	10 (9.9)	5 (4.8)
**All headache** ^**2**^	2,279	670 (29.4)	42 (6.3)	46 (6.8)

^1^Values reflect that not all pupils responded to every enquiry; ^2^includes unclassified headache

Overall, 7.9% of parents reportedly missed work at least once in 4 weeks because of their son’s or daughter’s headache (Table 6). The risk of parental work loss varied with headache type, being higher for migraine (10.5%) than for TTH (6.9%; *p* < 0.05) and UdH (4.1%; *p* < 0.001), but much higher for pMOH (26.8%) than for any of these (*p* < 0.01). The risk was also age-related (Table 6; *p* < 0.05 for all headache and migraine; *p* < 0.001 for pMOH), but in the opposite direction (lower for parents of adolescents) to other variables trending with age.

### Emotional impact and quality of life (QoL)

UdH had the least emotional impact (*p* < 0.001 vs. each other headache type), followed by TTH (also *p* < 0.001 vs. each other headache type) (Table 7). While pMOH and other H15 + were not significantly different from each other, both had greater impact (*p* < 0.001) than all episodic headaches (Table 7).

While QoL scores were not normally distributed, they proved quite sensitive to headache (Table 7): all types had significant impact (*p* < 0.001) despite a mean of only 27.8/36 among those with no headache. Again, the impact of UdH was least, followed by those of TTH and migraine; pMOH had greatest impact (*p* < 0.001 vs. UdH and TTH; *p* < 0.05 vs. migraine).

**Table 6:** Parents’ lost work in the preceding 4 weeks, by pupils’ headache type and overall, and by age group of pupil

Headache type	Pupils affected *n*	Headache frequency (from Table 1) days/4 weeks	Parent missed work *n* (% of pupils with headache type)
**Episodic headaches**			
Migraine	910	3.7	94 (10.5)
children	243	2.9	34 (14.3)
adolescents	667	4.0	60 (9.1)
Tension-type headache	435	2.7	30 (6.9)
children	130	1.8	12 (9.3)
adolescents	305	3.0	18 (5.9)
Undifferentiated headache	750		30 (4.1)
children	287	1.8	11 (3.9)
adolescents	463		19 (4.2)
**Headache on ≥ 15 days/month**			
Probable medication-overuse headache	41	20.9	11 (26.8)
children	5	23.2	5 (100)
adolescents	36	20.5	6 (16.7)
Other headache on ≥ 15 days/month	121	18.5	11 (9.1)
children	14		3 (21.4)
adolescents	107		8 (7.5)
**All headache** ^**1**^	2,301	3.9	179 (7.9)
children	703		65 (9.4)
adolescents	1,598		114 (7.2)

^1^Includes unclassified headache

## Discussion

This study has added data on attributed burden to the prevalence estimates previously reported among children and adolescents in Iran [7]. They are the first such data from Eastern Mediterranean Region. The following were the principal findings. Overall mean headache frequency was 3.9 days/4 weeks. With mean duration of 1.8 h, estimated mean pTIS was 1.1% (1.4% for migraine, 16.5% for pMOH) and headache-attributed lost health at individual level was 0.6% for migraine and 3.7% for pMOH. Symptomatic medication was reportedly taken on fewer than half of headache days (mean 1.6 days/4 weeks). Lost school time because of headache averaged 2% overall, but 11-fold more (22%) for pupils with pMOH, while, for most headache types, days of limited activity were several-fold more than days lost from school (45% for pMOH). Almost one in 12 parents (7.9%) also missed work.

**Table 7:** Emotional impact and quality of life, by headache type and overall

Headache type	Emotional impact scale 0-18^1^ (high adverse)		Quality-of-life scale 0-36^1^ (low adverse)	
	*n* ^2^	Score (mean ± SD)	*n* ^2^	Score (mean ± SD)
No headache	-	-	858	27.8 ± 5.0
**Episodic headaches**				
Migraine	876	6.8 ± 2.9	826	21.1 ± 6.2
Tension-type headache	415	5.4 ± 2.6	401	23.8 ± 5.7
Undifferentiated headache	704	4.6 ± 2.6	679	25.4 ± 5.8
**Headache on ≥ 15 days/month**				
Probable medication-overuse headache	41	9.6 ± 3.5	36	16.5 ± 6.3
Other headache on ≥ 15 days/month	116	8.0 ± 2.6	113	18.0 ± 5.7
**All headache**	2,195	5.9 ± 3.0		

^1^See text for explanation; ^2^values reflect that not all pupils responded to both enquiries

For the episodic headaches, therefore, the symptom burden of headache among these age groups appeared not to be highly onerous, only migraine (0.16%) being responsible for > 0.1% of lost health across the entire population of children and adolescents. Probably reflecting this, and in particular the reported short durations (overall mean of 1.8 h), medication days for the episodic headaches were fewer than headache days. This was notably true also for other H15+: despite a longer mean headache duration of 2.9 h, symptomatic medication was reportedly taken on only 4.3 of 18.5 headache days per 4 weeks. Yet this raises concern, because it nonetheless represents use of medication more than once a week on average by 3% of pupils (this being the prevalence of other H15 + in these age groups [7]).

For pMOH, however, medication days (18.0) were not significantly fewer than headache days (20.9). Somewhere, therefore, a behavioural change had occurred. With regard to this, it should be noted that the prevalence of pMOH is on a steep upward trajectory with increasing age, from 0.4% in children to 1.9% in adolescents [7]. Investigating the course (and causes) of this among young people may be easier than among adults, in whom medication overuse is often entrenched, the course (and causes) inextricable from the history [15].

If symptom burdens might be relatively modest, at least for the episodic headaches, the reported consequential burdens cannot be lightly regarded. Lost school time, with a mean of 0.4 days/4 weeks (2%) for migraine (considerably more for some pupils, as evidenced by the SD of 1.0), is likely to have some adverse impact on education. Those with pMOH, carrying a much higher lost-health burden of 3.7%, reportedly also lost > 1 day/week of schooling on average, which must be highly damaging to their education while also jeopardising their lifetime prospects (cumulative burden [16]). These findings were based on recall, but supported by similar findings in association with HY, with school absence objectively recorded. Notably, while age-related differences were minor for most headache types, adolescents reported substantially fewer school days lost to pMOH (3.2) than children (11.7). An explanation readily suggests itself – better coping by adolescents with highly frequent headache – but it should be noted that only five children contributed to this analysis. The differential also existed, but was less marked, in days of limited activity (11.7 vs. 18.6), so another possible explanation, if these differences were real, was greater concern among adolescents than children about the impact on schooling, and greater reluctance, therefore, to miss school.

Other activities, outside school – far from unimportant in these age groups – fared substantially worse than schooling, being limited, overall, on 6.4% of all days, on 45% of all days for pMOH, and on 25% for other H15+. A day of limited activity may not imply that the whole day was lost, but these numbers are nonetheless indicative at least of life diminution, and, for those with pMOH or other H15+, of substantial life impairments. The emotional impact and QoL scores reflected this. The former, on a scale of 0–18 generated by six headache-relevant questions from KINDL® [23] (principally addressing concentration, mood, fear of headache and coping with it), are not intuitively meaningful but showed a clear gradient of adverse impact: pMOH having the greatest (9.6/18), followed by other H15+, migraine and TTH, and UdH (4.6/18) having the least – all as expected. QoL scores, similarly, are not intuitively meaningful, but here proved highly sensitive to headache: 77% in pupils without headache, 71% for those with UdH, 59% for migraine and as low as 46% for pMOH and 50% for other H15+.

A further, and important, consequential burden was lost parental work, reportedly affecting 7.9% of parents. We did not attempt to quantify these losses with any precision, or establish what proportion of parents were in paid work, not expecting younger pupils in particular to be reliably aware of these details. Nevertheless, this finding – almost one in 12 parents affected in this way (one in four [26.8%] parents of pupils with pMOH) – is evidence of a substantial and to some extent hidden impact [16], itself a potential cause of further consequences for parents and family. Parental work losses were age-related, but, notably, in the opposite direction to other variables trending with age. Presumably, since the same has been found elsewhere [39], this reflected less dependence upon parents among adolescents than among children despite, for the former, both greater probability of headache and, for migraine and TTH, greater pTIS.

Almost 20% of Iran’s 88 million population are aged 6–18 years [29], with the vast majority attending school [30]. These findings, therefore, should be factored into educational policy in Iran. With regard to health care, Iran’s constitution, since the 1979 revolution, has established that “every Iranian citizen has a right to enjoy the highest attainable level of health” [31], asserting an obligation within the country of universal health care. Despite substantial progress towards fulfilling this for communicable, maternal and neonatal diseases, Iran’s health policy has been less successful with regard to non-communicable diseases [32]. Since headache disorders are largely treatable [33], these headache-attributed burdens among young people are evidence of deficiencies in health care that allow them to persist. Iran is by no means alone in this: in 2011, the World Health Organization along with the Global Campaign against Headache observed similar failures in countries throughout the world [34]. Solutions exist [35], which Iran’s health services are well structured to accommodate [36], and which, if fully implemented, might generate a positive return on investment [37, 38].

The only country that has so far produced similar published data is Lithuania [39]. More will be forthcoming as the Global Campaign completes its programme of schools-based studies around the world [17, 19]. Meanwhile, the findings of that study [39] were very much in line with ours from Iran. This is important, because it suggests the enquiry was robust, despite the difficulties in formulating questions for, and obtaining reliable responses from, children as young as six. Formal validations in young people are both ethically challenging and in practice difficult to conduct.

The strengths of this study are as noted previously [7]: its use of standard methodology [17], its nationwide sampling and its adequate sample size [20] with a non-participating proportion of only 3.4%. The sample was well balanced for gender, while the relative under-sampling of children (40.3%) was not problematic since their number (1,308) remained high. The principal limitation – already referred to – lay in the necessary dependence on the uncertain reliability of young people’s comprehension (mitigated here by asking teachers to mediate the enquiry), on their willingness and ability to respond (both encouraged by conducting the enquiry in class), and on their recall over 4 weeks. Inevitably there was some imprecision. In particular, usual duration of headache episodes was reported categorically (< 1, 1–2, 2–4 or > 4 h). In taking the mid-points of each, we assumed, conservatively, a range of 4–12 h for the last, since longer episodes are unusual in children [4, 22]. As a means of circumventing this limitation, enquiry of parents, rather than children gathered in school, would be logistically far more difficult and much more demanding of resources. Perhaps more pertinently, it might be even less reliable [40].

## Conclusions

Headache among children and adolescents in Iran generates symptom burdens that do not appear to be heavy for the majority, principally because short durations lead to low pTIS, but they are notably heavy for some. Accordingly, there are substantial consequential burdens, particularly for the 1.1% with pMOH and, somewhat less so, for the 3.0% with other H15+, who suffer educational disturbances and potentially major life impairments. Educational and health policies in Iran would do well to take note of these findings.

## Data Availability

The data are held on file at the University of Mersin. Once analysis and publications are completed, they will be freely available for non-commercial purposes to any person requesting access in accordance with the general policy of the Global Campaign against Headache.

## Abbreviations

ANOVA: Analysis of variance
DW: Disability weight
GBD: Global Burden of Disease (study)
H15+: Headache on ≥ 15 days/month
HARDSHIP: Headache-Attributed Restriction, Disability, Social Handicap and Impaired Participation (questionnaire)
HY: Headache yesterday
ICHD: International Classification of Headache Disorders
MOH: Medication-overuse headache
pMOH: probable MOH
pTIS: Proportion of time in ictal state
QoL: Quality of life
SD: Standard deviation
TTH: Tension-type headache
UdH: Undifferentiated headache
YLD: Year lived with disability
